# ASF Vaccine Candidate ASFV-G-∆I177L Does Not Exhibit Residual Virulence in Long-Term Clinical Studies

**DOI:** 10.3390/pathogens12060805

**Published:** 2023-06-06

**Authors:** Manuel V. Borca, Elizabeth Ramirez-Medina, Ediane Silva, Ayushi Rai, Nallely Espinoza, Lauro Velazquez-Salinas, Douglas P. Gladue

**Affiliations:** 1 Plum Island Animal Disease Center, ARS, USDA, Greenport, NY 11944, USA; elizabeth.ramirez@usda.gov (E.R.-M.); ediane.silva@usda.gov (E.S.); ayushi.rai@usda.gov (A.R.); nallely.espinoza@usda.gov (N.E.); lauro.velazquez@usda.gov (L.V.-S.); 2 Oak Ridge Institute for Science and Education (ORISE), Oak Ridge, TN 37830, USA

**Keywords:** ASFV, virus virulence, long-term clinical studies, live-attenuated vaccines, ASFV-G-∆I177L

## Abstract

African swine fever (ASF) is an important disease in swine currently producing a pandemic affecting pig production worldwide. Except in Vietnam, where two vaccines were recently approved for controlled use in the field, no vaccine is commercially available for disease control. Up to now, the most effective vaccines developed are based on the use of live-attenuated viruses. Most of these promising vaccine candidates were developed by deleting virus genes involved in the process of viral pathogenesis and disease production. Therefore, these vaccine candidates were developed via the genomic modification of parental virus field strains, producing recombinant viruses and reducing or eliminating their residual virulence. In this scenario, it is critical to confirm the absence of any residual virulence in the vaccine candidate. This report describes the assessment of the presence of residual virulence in the ASFV vaccine candidate ASFV-G-∆I177L in clinical studies conducted under high virus loads and long-term observation periods. The results demonstrated that domestic pigs intramuscularly inoculated with 10^6^ HAD_50_ of ASFV-G-∆I177L did not show the presence of any clinical sign associated with ASF when observed daily either 90 or 180 days after vaccination. In addition, necropsies conducted at the end of the experiment confirmed the absence of macroscopic internal lesions associated with the disease. These results corroborate the safety of using ASFV-G-∆I177L as a vaccine candidate.

## 1. Introduction

African swine fever virus (ASFV), the causative agent of ASF, is a structurally complex virus with a large DNA double-stranded genome encoding more than 150–160 genes [1]. The disease is currently present as a pandemic, affecting swine production worldwide. Historically secluded in Africa, after an initial outbreak in the Republic of Georgia in 2007, the disease quickly spread to central and eastern Europe, reaching China in 2018 and later spreading throughout southeast Asia [2,3,4]. In addition, the disease was reported in Malaysia and the Hispaniola Islands in 2021, constituting the first report of ASF in the western hemisphere since the 1970s [5]. Continued outbreaks of ASF in affected countries are causing devastating economic losses to swine production as well as a shortage in worldwide protein availability [6].

Live-attenuated vaccine candidates have been developed via genetic manipulation, inducing protection against the current pandemic strain [7,8,9,10,11]. Recombinant viruses, developed by removing viral genes associated with virulence, efficiently protect pigs against infection with the homologous virulent parental virus [7,8,9,10,11,12,13,14,15]. With the exception of ASFV-G-∆I177L (available in Vietnam), there is no commercially available vaccine to prevent ASF; therefore, disease control is based on the elimination of affected animals and following strict quarantine regulations. Vietnam licensed the first commercial genetically modified live vaccine in 2022, the vaccine strain ASFV-G-∆I177L [9]. ASFV-G-∆I177L was developed by partially removing the I177L gene from the genome of the highly virulent strain Georgia (ASFV-G). ASFV-G-∆I177L has been shown to effectively protect pigs against infection with ASFV-G even when used in doses as low as 10^2^ HAD_50_. In addition, ASFV-G-∆I177L induces sterile protection when used in doses of 10^4^ HAD_50_ or higher and, importantly, lacks residual virulence even in doses as high as 10^6^ HAD_50_ when tested in short-term studies [9,16]. The efficacy profile of commercially produced stocks of ASFV-G-∆I177L were recently reported on regarding their use against Vietnamese field strains using animals of different genetic backgrounds [16].

A critical issue in the use of live-attenuated vaccine strains is the evaluation of the absence of residual virulence in the vaccine virus. This is particularly important in ASFV, where residual virulence is a recurrent issue in natural as well as genetically modified attenuated strains [17,18,19]. In this regard, the appearance of chronic clinical forms of the disease has been reported several times as the main example of residual virulence. We report here an evaluation of the potential presence of residual virulence in the ASFV-G-∆I177L vaccine in long-term clinical studies performed using high doses of the virus. The results showed the absence of clinical ASF disease in vaccinated animals evaluated for up to 180 days, confirming the safety of ASFV-G-∆I177L as a vaccine candidate.

## 2. Materials and Methods

### 2.1. Viruses and Cells

The vaccine candidate ASFV-G-∆I177L was produced via recombination, as previously described [8]. The recombinant virus contains a partial gene deletion that interrupts the expression of the I77L protein in the virulent ASFV strain Georgia 2007 [8]. Cell cultures of primary swine macrophages were used to assess the presence of ASFV-G-∆I177L in blood samples collected during this experiment. These cell cultures were prepared as previously described [8]. The assessment was conducted on 96-well plates containing a total of 1 × 10^6^ cells per plate, with the presence of infected cells evaluated using hemadsorption (HA). Final viral titers were determined as previously described [8].

### 2.2. Evaluation of ASFV-G-∆I177L Virulence in Long-Term Experiments on Domestic Pigs

The virulence of ASFV-G-∆I177L was evaluated in 15–20 kg commercial Yorkshire cross-breed swine. Two independent experiments were performed where animals intramuscularly (IM) inoculated with 10^6^ HAD_50_ of ASFV-G-∆I177L were daily monitored for clinical signs associated with ASF (fever, anorexia, depression, diarrhea, purple skin discoloration, staggering gait, and cough). In the first experiment, 5 pigs were inoculated with ASFV-G-∆I177L while another 5 animals were mock-inoculated with a macrophage culture medium. In the second experiment, 10 animals were inoculated with ASFV-G-∆I177L, while 5 animals were mock-inoculated. All animals were observed for 90 and 180 days, respectively. Blood samples were collected at various days post-inoculation as depicted in the results section. All experiments were conducted under biosafety level 3 conditions at the Plum Animal Disease Center. Animal procedures were approved by the Institutional Animal Care and Use Committee under protocol 225.06-19-R_090716 (approved on 9 June 19).

### 2.3. Detection of ASFV-Specific Antibodies

The presence of antibodies against ASFV was determined using an in-house ELISA, as previously described [8]. Briefly, 96-well plates were coated with 1 µg per well of infected or uninfected cell extracts produced in Vero cells. Plates were blocked with 10% skim milk and 5% normal goat serum. Multiple dilutions of each serum were evaluated against both infected and uninfected cell antigens. The detection of specific antibodies against ASFV was conducted using an anti-swine IgG-horseradish peroxidase conjugate and SureBlue Reserve peroxidase substrate. Finally, plates were read at OD630 nm using a plate reader (ELX800). The titer of different serums represents the inverse log_10_ of the highest serum dilution where the readings of the tested sera at least duplicated the readings of the mock-infected sera.

## 3. Results and Discussion

### Assessment of the Presence of the Residual Virulence of ASFV-G-∆I177L in Long-Term Experiments on Domestic Pigs

To assess the potential presence of the residual virulence of ASFV-G-∆I177L in domestic pigs, the effect of inoculation using high doses of the virus was evaluated in two long-term experiments, evaluating the appearance of the clinical signs of the disease in animals observed for 3 or 6 months after inoculation with the vaccine virus.

In the first experiment, a group of five pigs was intramuscularly (IM) inoculated with 10^6^ HAD_50_ of ASFV-G-∆I177L. The presence of clinical signs associated with ASF was evaluated daily over an observational period of 90 days. A control group of five animals with the same characteristics as the ones inoculated with ASFV-G-∆I177L was mock-inoculated with a macrophage culture medium.

**Figure 1:**
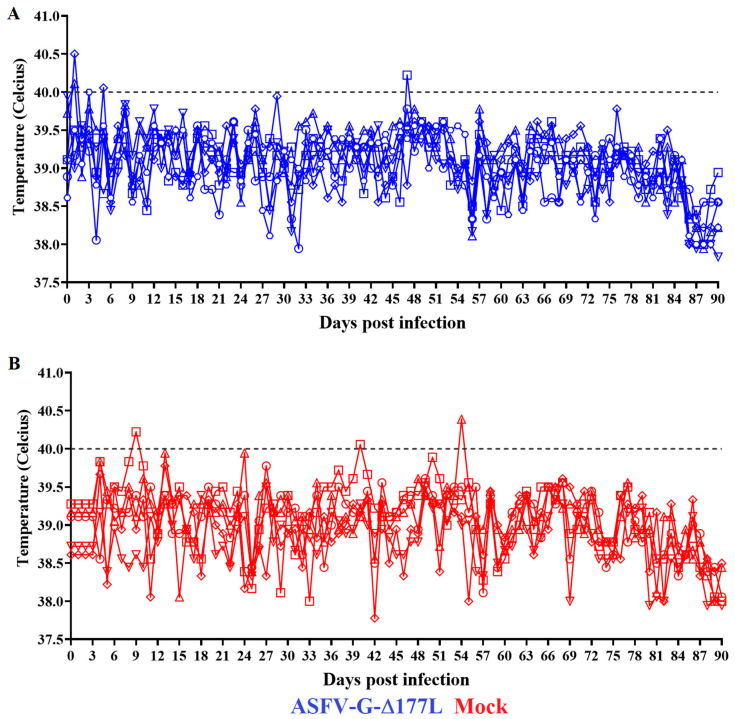
Evolution of body temperature in animals IM-inoculated with 10^6^ HAD_50_ of ASFV-G-∆I177L (**A**) or mock-inoculated (**B**) and observed for 90 days post-inoculation. Symbols represent specific animals at each experimental group.

The clinical evaluation performed on a daily basis demonstrated the complete absence of any significant abnormality or clinical signs that may be associated with the disease. The animals remained clinically normal (bright, alert, and responsive) during the three months of observation. No differences were observed in this group of animals in terms of clinical observation related to that of the mock-inoculated group of animals. The evolution of the daily body temperatures of the animals inoculated with ASFV-G-∆I177L demonstrated that, with the exception of a few isolated cases, all animals presented values lower than 40 °C for the three months after vaccination, showing body temperature readings similar to the mock-inoculated control pigs (Figure 1A,B).

At the end of the experiment, all animals from both groups were euthanized, and necropsies were conducted in order to detect the presence of internal macroscopical lesions usually found in ASF-infected animals. No macroscopical lesions that could be associated with ASF were detected in the ASFV-G-∆I177L-inoculated animals, whose internal organ conditions were indistinguishable from those observed in animals from the mock-inoculated group.

**Figure 2:**
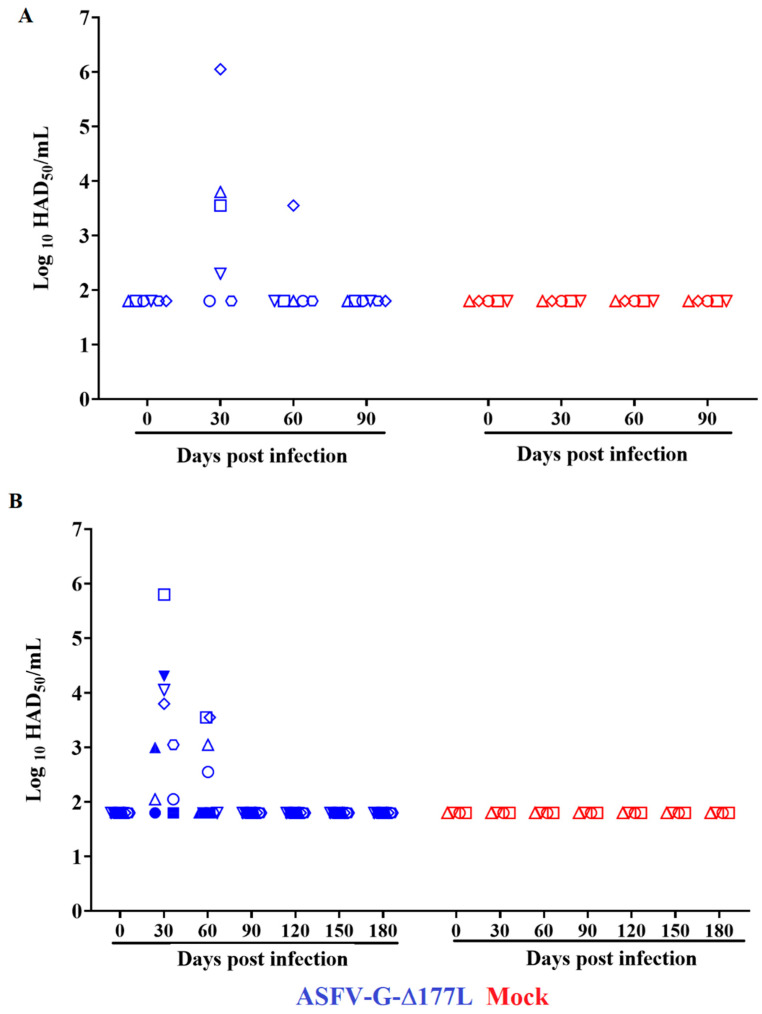
Viremia titers in animals IM-inoculated with 10^6^ HAD_50_ of ASFV-G-∆I177L and observed for either (**A**) 90 or (**B**) 180 days post-inoculation. Virus titers are expressed as log10 HAD_50_/mL of blood. Sensitivity of detection: ≤1.8 HAD_50_/mL. Symbols represent specific animals at each experimental group.

The evolution of viremia in the animals inoculated with ASFV-G-∆I177L indicates the expected kinetics based on previous results [9,16,20]. All animals presented extended viremias within the first month of inoculation, showing titers ranging from undetectable to 10^6.05^ HAD_50_/mL by day 30 post-inoculation (PI) (Figure 2A). Viremias tested on day 60 PI demonstrated that all animals remained negative except one, which had a titer of 10^3.55^ HAD_50_/mL. At 90 days PI, the virus in all the animals in the group was undetectable in their blood (titers ≤ 10^1.8^ HAD_50_/mL).

**Figure 3:**
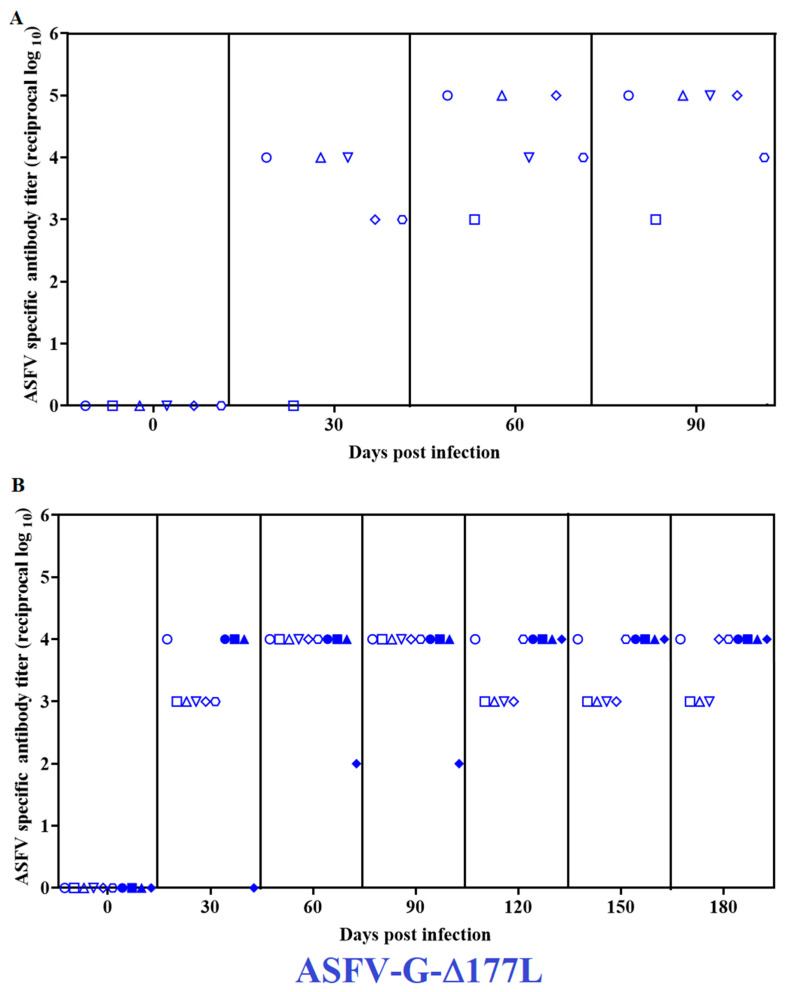
ASFV-specific antibody titers detected in pigs IM-inoculated with 10^6^ HAD_50_ of ASFV-G-∆I177L and observed for either (**A**) 90 or (**B**) 180 days post-inoculation. Symbols represent different animals in the experimental group.

The detection of ASFV-specific antibody responses was performed with an in-house-developed direct ELISA using extracts of cell cultures infected with ASFV as an antigen. At 30 days PI, all animals presented high antibody titers, ranging from 10^3^ to 10^4^/mL (Figure 3A). Antibody titers increased when measured at 60 days PI, reaching titers of 10^3^–10^5^/mL that remained at those values until the end of the experimental period, at 90 days PI.

The second experiment performed was aimed at evaluating the potential presence of the residual virulence of ASFV-G-∆I177L over a 180-day observational period. For that purpose, a group of ten pigs was IM-inoculated (also with 10^6^ HAD_50_ of ASFV-G-∆I177L), while a control group of five animals was mock-inoculated with a macrophage culture medium. Both groups were monitored daily for 180 days, watching for the presence of clinical signs associated with ASF.

**Figure 4:**
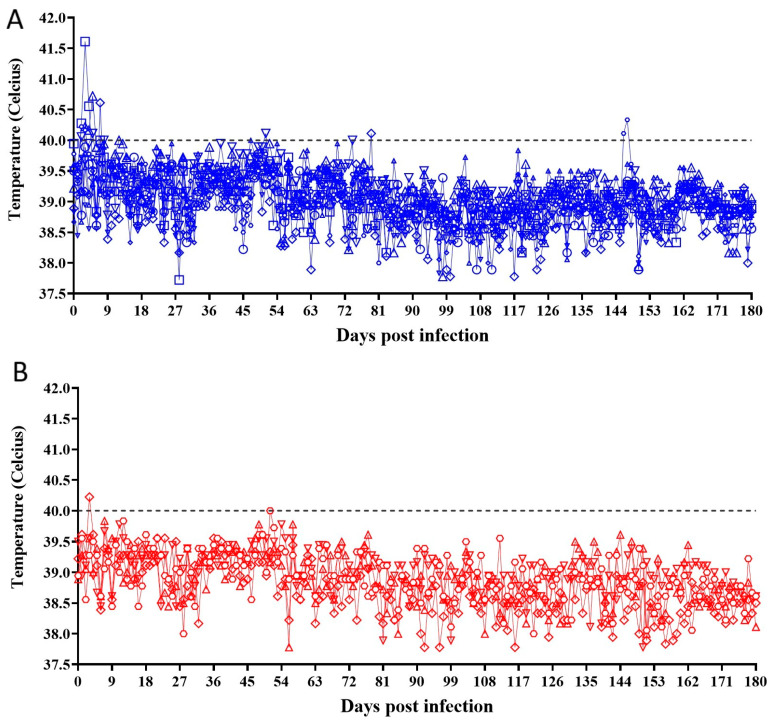
Evolution of body temperature in animals IM-inoculated with 10^6^ HAD_50_ of ASFV-G-∆I177L (**A**) or mock-inoculated (**B**) and observed for 180 days post-inoculation. Symbols represent specific animals at each experimental group.

No clinical signs were detected in any of the animals inoculated with ASFV-G-∆I177L during the daily evaluation performed for 6 months after inoculation. No differences compared with the clinical data obtained from the group of mock-inoculated animals were observed. The body temperature kinetics of animals inoculated with ASFV-G-∆I177L indicate that all animals showed values within the normal range (always below 40 °C) during the 180 days PI, with a few exceptions, in particular, at very early days after inoculation in animals inoculated with ASFV-G-∆I177L. Animals in the mock-inoculated group presented similar body temperature values to the ASFV-G-∆I177L-inoculated ones (Figure 4).

As in the 90-day experiment, all animals from both groups were euthanized and necropsied, looking for the presence of macroscopical lesions associated with ASF-infected animals. No alterations were found in the animals inoculated with ASFV-G-∆I177L or in the mock-inoculated animals.

The viremia kinetics of ASFV-G-∆I177L-inoculated animals presented, as in the previous experiment, variable virus titers during the first month PI, with values ranging between 10^2.05^ and 10^6.8^ HAD_50_/mL by day 30 PI (Figure 2B). At day 60 PI, viremia titers showed low titers ranging between undetectable and 10^3.55^ HAD_50_/mL. The virus became undetectable in the blood of all the animals by day 90 PI and remained at that level in all animals when tested 120-, 150-, and 180-days PI.

The presence of virus-specific antibodies showed, as in the first experiment, serum titers ranging from 10^3^ to 10^4^/mL (Figure 2B) at day 30 pi. They remained at similar levels in all animals when tested at time points 60-, 90-, 120-, 150-, and 180-days PI. Antibody titers remained at background levels in all mock-inoculated animals at all time points tested during the 180-day experiment.

In domestic swine, ASFV produces a clinical form of the disease that ranges from a subclinical to a highly lethal presentation. The disease outcome heavily depends on the active virus strain [1,2]. Therefore, residual virus virulence can present a variety of clinical forms of the disease. ASFV-G-∆I177L has been tested in the absence of residual virulence in terms of causing an acute form of the disease [8,9,16,20], but data demonstrating complete attenuation in long-term experiments were not assessed until now. Natural attenuated strains usually present residual virulence in a chronic form of the disease, characterized by joint inflammation and skin lesions that appear several weeks after infection [1,2,17,18,19].

Taken together, the results presented here indicate that the ASFV-G-∆I177L vaccine candidate lacks residual virulence even when tested in long-term studies on animals that have been inoculated with relatively high doses of the virus. It should be noted that the dose tested in this study is 10,000 times higher than the minimal protective doses used in experimental conditions [8,16,20]. Currently, ASFV-G-∆I177L is being actively tested in field conditions at doses of 10^2.6^ HAD_50_ via the IM route. Therefore, the circumstances used in this report to test the residual virulence of ASFV-G-∆I177L far exceed the conditions that the vaccine virus will be used in during field vaccinations. However, we should be cautious in directly extrapolating the results reported here to animals of different ages, reproductive statuses, or genetic backgrounds.

## 4. Conclusions

The data presented here, along with those from previously published studies [7,8,16,20], indicate that ASFV-G-∆I177L lacks residual virulence even when used at relatively high doses (approximately 10,000 times the minimal protective dose) and evaluated during long observational periods. These results confirm the safety of the use of ASFV-G-∆I177L as a vaccine candidate.

## Data Availability

The data are contained within the article.
